# The Southwark Guardians and Guy's Hospital

**Published:** 1913-05-31

**Authors:** 


### THE SOUTHWARK GUARDIANS AND GUY'S HOSPITAL.

### Report by the Distribution Committee of King Edward's Hospital Fund.

We have received from the Honorary Secretaries of the King's Fund the following report of the King's Fund on the differences between Guy's Hospital and the Southwark Guardians. The extracts from this report which have appeared in the press were not furnished by the Fund and are incomplete.

The Distribution Committee have considered the statement received from the Southwark Guardians, dated November 14, 1912, with reference to Guy's Hospital, which was referred to them for the purpose of ascertaining whether there was anything in it which called for fresh comment or action on the part of the Fund.

They have also had before them a summary of the cases previously considered by the Fund—viz. three cases in 1903 reported by the Guardians to the Executive Committee of the Fund; one case reported by the Guardians to the Executive in 1905, when the Fund expressed itself as satisfied with the explanation given by Guy's; and two cases which came to the notice of the Fund in November 1911 through press reports of the inquests, including the case of Mrs. Mallandain, into which the Distribution Committee themselves inquired.

The statement raises an old question—viz. the reference from Guy's to the Guardians of cases for which Guy's have no room or which they consider more suitable for the Poor-Law infirmary than for the hospital.

The explanation of Guy's is that their number of beds is limited, so that they cannot take in all kinds of cases or even all urgent cases; that it is the duty of the Guardians to provide for the sick poor for whom there is no accommodation in the hospitals; and that the difficulty in urgent cases arises because the Southwark Infirmary is four miles off and the Guardians will not provide an emergency receiving house.

The Guardians argue that their duties are confined to the treatment of the "necessitous poor"; that Guy's ought to take in all cases above that class and particularly urgent cases; that the workhouse already provides adequately for local necessitous poor; and that to provide a receiving house would encourage Guy's to send them cases belonging to other Unions.

The Distribution Committee reported in the case of Mrs. Mallandain that they considered it desirable that additional provision should be made at Guy's for emergency cases, especially at night, and they adhere to that view. They suggested at the time that two or more surgery beds might be kept free every night for emergencies. To this Guy's replied that until they were in a position to build new accident wards and a new casualty department they could not make better arrangements than now exist. The hospital now states that the scheme of extension upon which they are at present engaged will, when completed some years hence, afford space for a female accident ward, if funds for its upkeep are then available.

The Distribution Committee consider, however, that as the infirmary is so far away, the Guardians ought to establish a reception station in the neighbourhood where it is required, since they have the power to do so. The Committee think that it would be absolutely wrong for Guy's to overcrowd its surgical wards with emergency cases; the same observation applying, though not so strongly, to the medical wards. In their opinion a voluntary hospital should not be expected to admit incurable cases.

The fact that Guy's attracts patients resident outside the Union, who subsequently become chargeable to the Union, is no reflection on Guy's, and the Guardians have their own remedy by discovering the legal settlement of such persons and making them chargeable accordingly.

